# Data from computational analysis of the peptide linkers in the MocR bacterial transcriptional regulators

**DOI:** 10.1016/j.dib.2016.08.064

**Published:** 2016-09-05

**Authors:** Sebastiana Angelaccio, Teresa Milano, Angela Tramonti, Martino Luigi Di Salvo, Roberto Contestabile, Stefano Pascarella

**Affiliations:** a Dipartimento di Scienze biochimiche “A. Rossi Fanelli”, Sapienza Università di Roma, 00185 Roma, Italy; b Istituto di Biologia e Patologia Molecolari, Consiglio Nazionale delle Ricerche, 00185 Roma, Italy

**Keywords:** Linker peptide, Linker length, MocR regulators, Linker engineering, PdxR, GabR, Hydrophobicity, Flexibility, Residue propensity, Dyad propensity

## Abstract

Detailed data from statistical analyses of the structural properties of the inter-domain linker peptides of the bacterial regulators of the family MocR are herein reported. MocR regulators are a recently discovered subfamily of bacterial regulators possessing an N-terminal domain, 60 residue long on average, folded as the winged-helix-turn-helix architecture responsible for DNA recognition and binding, and a large C-terminal domain (350 residue on average) that belongs to the fold type-I pyridoxal 5′-phosphate (PLP) dependent enzymes such aspartate aminotransferase. Data show the distribution of several structural characteristics of the linkers taken from bacterial species from five different phyla, namely Actinobacteria, Alpha-, Beta-, Gammaproteobacteria and Firmicutes.

Interpretation and discussion of reported data refer to the article “*Structural properties of the linkers connecting the N- and C- terminal domains in the MocR bacterial transcriptional regulators*” (T. Milano, S. Angelaccio, A. Tramonti, M. L. Di Salvo, R. Contestabile, S. Pascarella, 2016) [1].

**Specifications Table**

**Table:** 

Subject area	*Biology*
More specific subject area	*Structural properties of linkers in the bacterial transcriptional regulators*
Type of data	*Table, graph, figure*
How data was acquired	*Databank searches. Computational analysis*
Data format	*Raw, filtered, analyzed*
Experimental factors	*Analyses were mostly carried out with Perl, Python and R scripts and software for structural bioinformatics*
Experimental features	*Linker sequences were extracted from multiple sequence alignments of MocR regulators. Computational analysis defined the residue and residue dyads propensities and the distribution of physicochemical properties in the linker sequences.*
Data source location	*UniProt, RefSeq*
Data accessibility	*Data is within this article. Linker sequence sets are available at*https://sites.google.com/a/uniroma1.it/pascarellalab/home/resources

**Value of the data**

• Data represent the description of the structural properties of the peptide linkers connecting the N- and C-terminal domains in the MocR bacterial regulators.
• Data provide researchers with a framework to select specific MocR for experimental characterization.
• Data provide a support to design experiments for the investigation of properties of specific MocR: for example, experiments of site-directed mutagenesis, deletions or insertions of linker regions.
• Data can help interpretation of experimental data obtained from MocR studies.
• Data provide a framework to derive rules for *de-novo* design of peptide linkers with desired properties.

## 1 Data

Results derived from computational analysis of the inter-domain sequences of the peptide linker connecting the N-terminal and the C-terminal domain of the bacterial transcriptional regulators of the subfamily MocR are herein reported. Data are shown as tables describing linker statistics such as residue and dyad composition propensities, predicted secondary structure frequency, and box-plots showing the distribution of several structural properties. Moreover, plots of length distributions of linkers from two specific MocR subgroups, namely PdxR and GabR, are also reported.

## 2 Experimental design, materials and methods

**Fig. 1:**
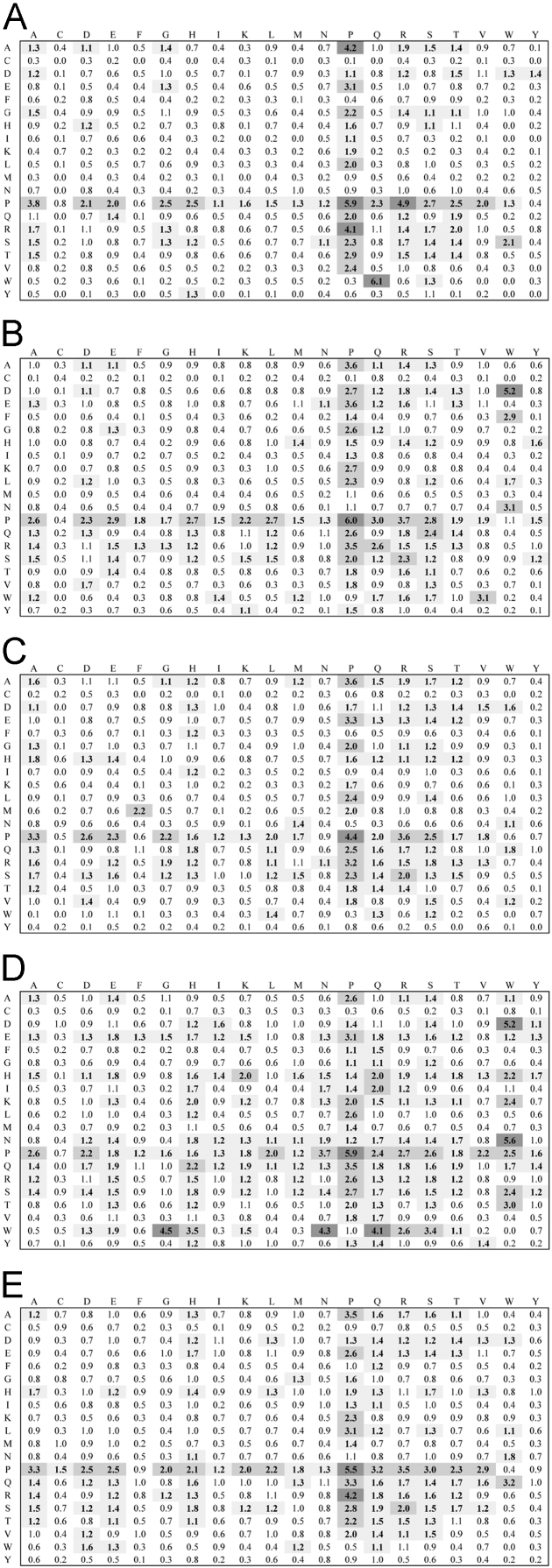
Dipeptide propensity for the entire set of linkers. Vertical and horizontal sides of each matrix indicate the N- and C-side residue of each dyad, respectively. Cells containing propensity values ≥1.1 and ≤1.99 or ≥2.0 and ≤3.99 or ≥4.0 are shaded with very light, light or dark grey respectively and numbers therein contained are boldfaced. A, B, C, D and E denote propensities for Actinobacteria, Alphaproteobacteria, Betaproteobacteria, Firmicutes and Gammaproteobacteria, respectively.

**Fig. 2:**
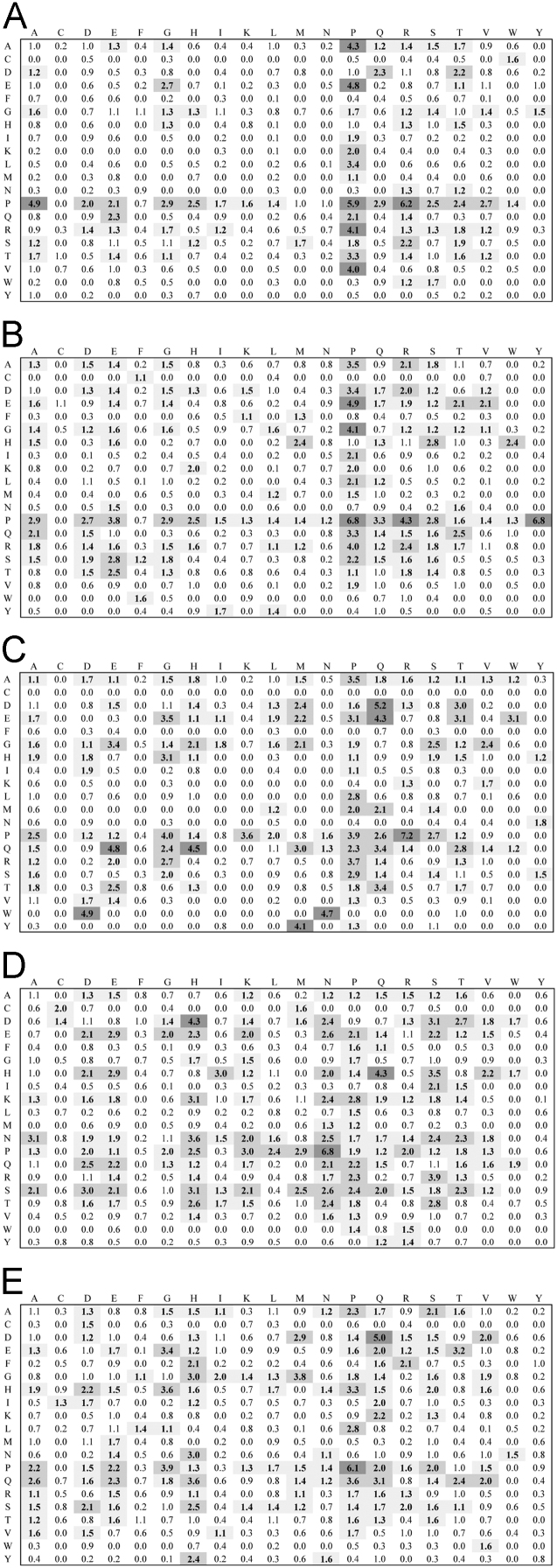
Dipeptide propensity for the 0–20 residue length linker set. Interpretation of figure refers to legend to Fig. 1.

**Fig. 3:**
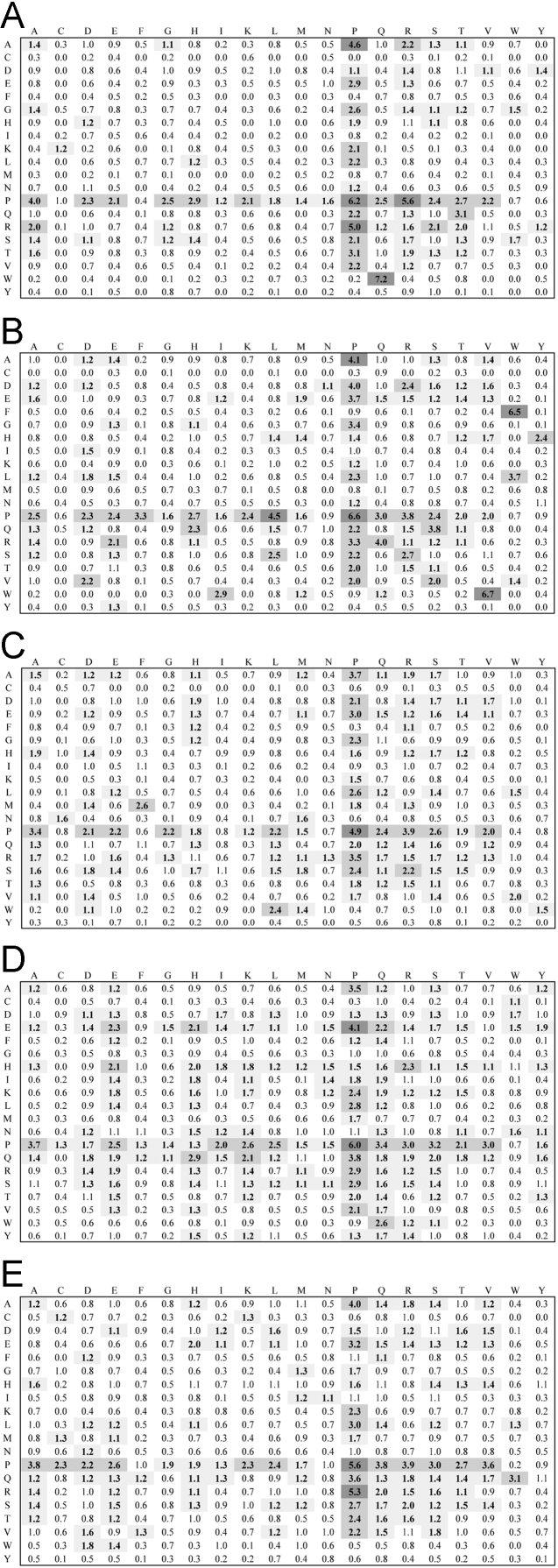
Dipeptide propensity for the 21–40 residue length linker set. Interpretation of figure refers to legend to Fig. 1.

**Fig. 4:**
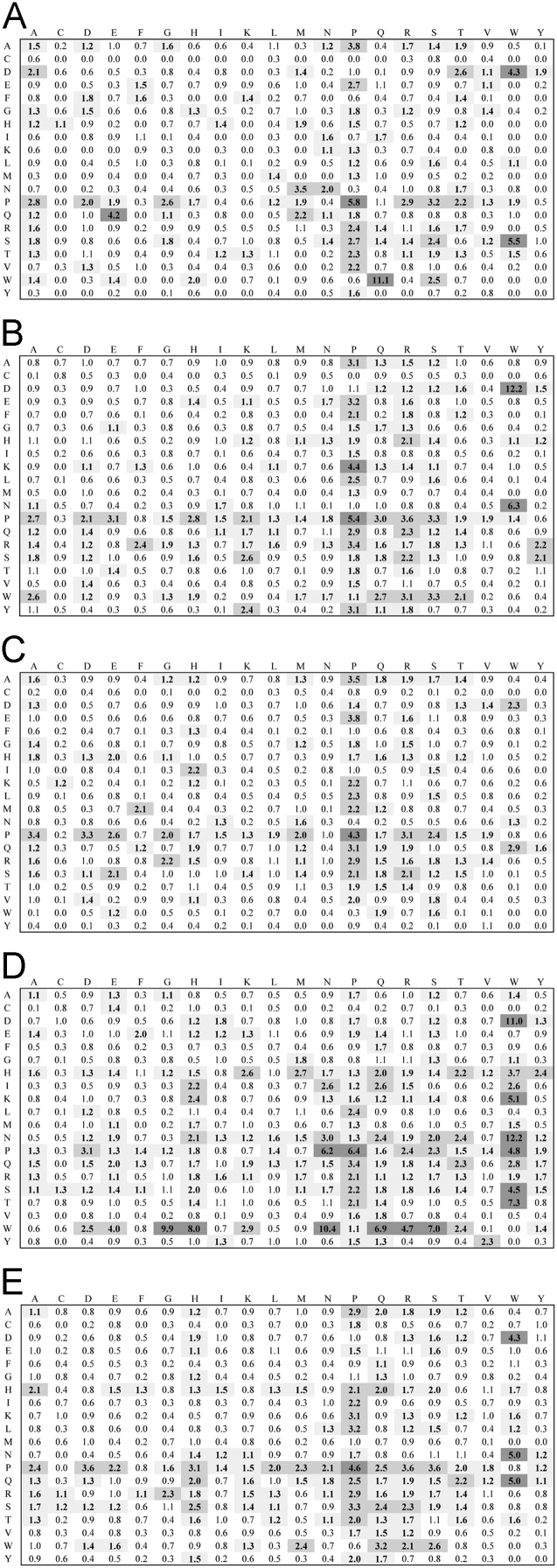
Dipeptide propensity for the 41–60 residue length linker set. Interpretation of figure refers to legend to Fig. 1.

**Fig. 5:**
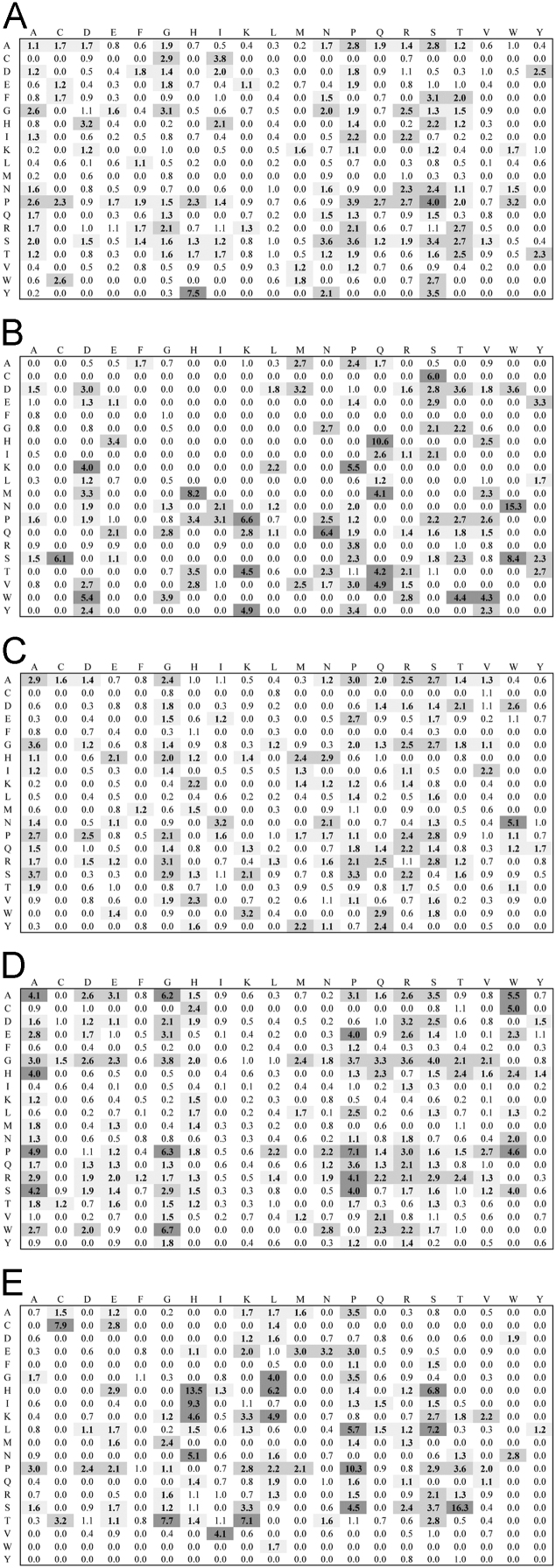
Dipeptide propensity for the 61–200 residue length linker set. Interpretation of figure refers to legend to Fig. 1.

**Fig. 6:**
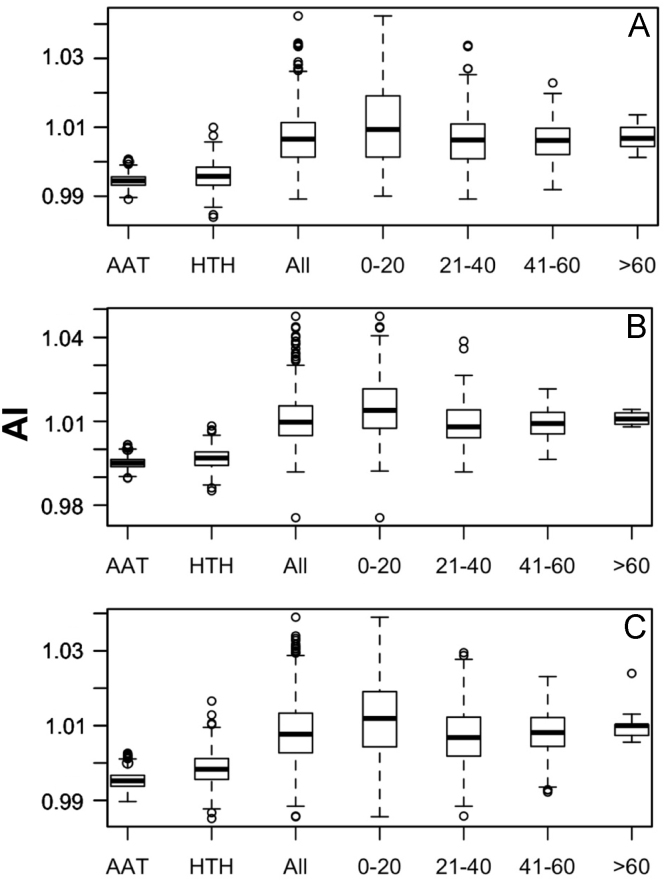
Box-plots of the distribution of the average linker flexibility (index #425 of Table 2 in [1] and code VINM940101 in AAindex [10]). Horizontal axis indicates the average flexibility distribution in the wHTH, AAT domains, in all linkers, and in linkers belonging to different length intervals: 0–20, 21–40, 41–60 and >60 residues. Y-axis reports the flexibility scale (label AI stands for Average Index). A, B, and C, denote Alphaproteobacteria, Betaproteobacteria, and Gammaproteobacteria, respectively.

**Fig. 7:**
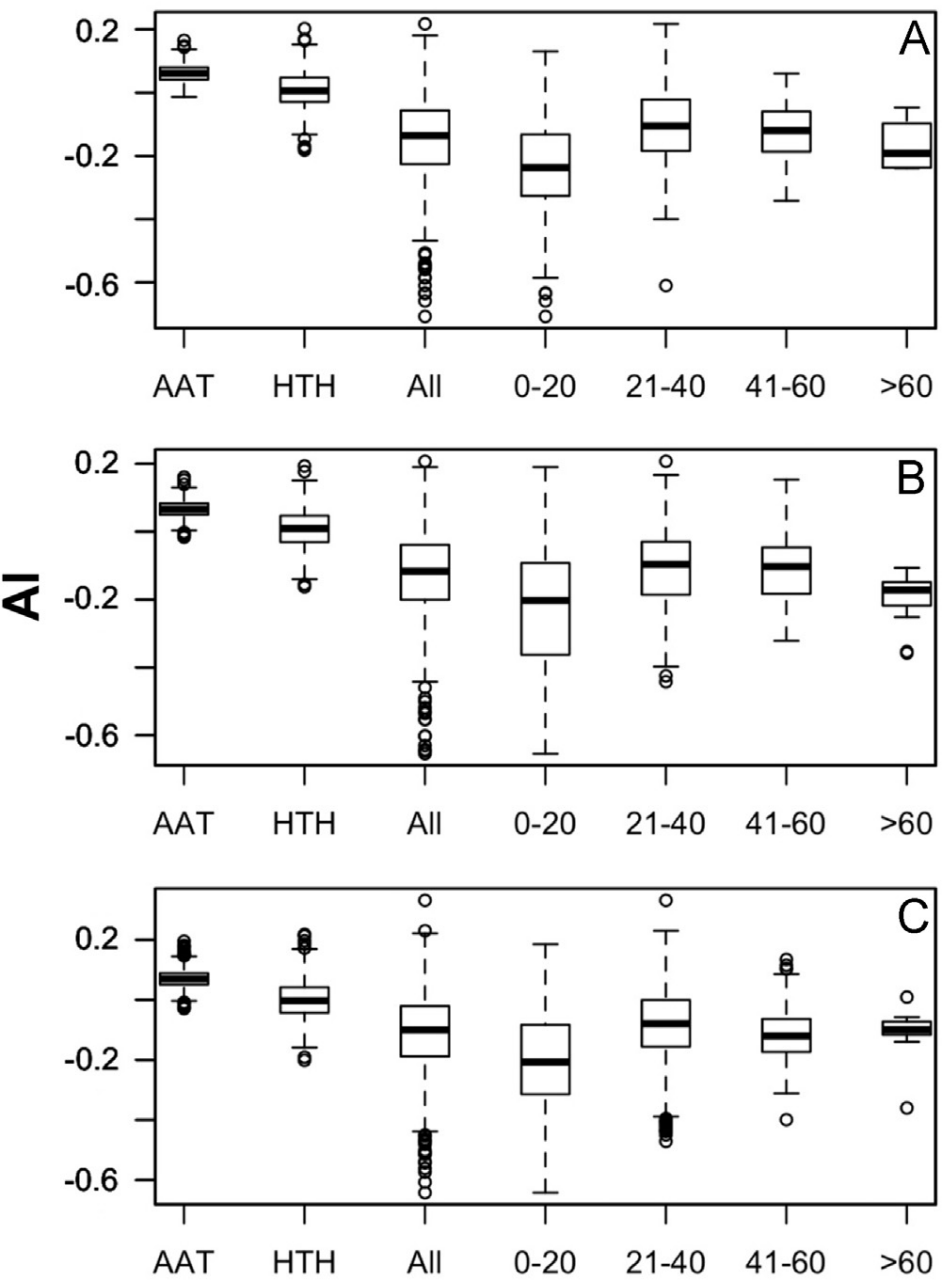
Box plots of the distribution of average linker hydrophobicity (index #58 of Table 2 in [1] and code CIDH920105 in AAindex [10]). For interpretation of plots, refer to Fig. 6 caption.

**Fig. 8:**
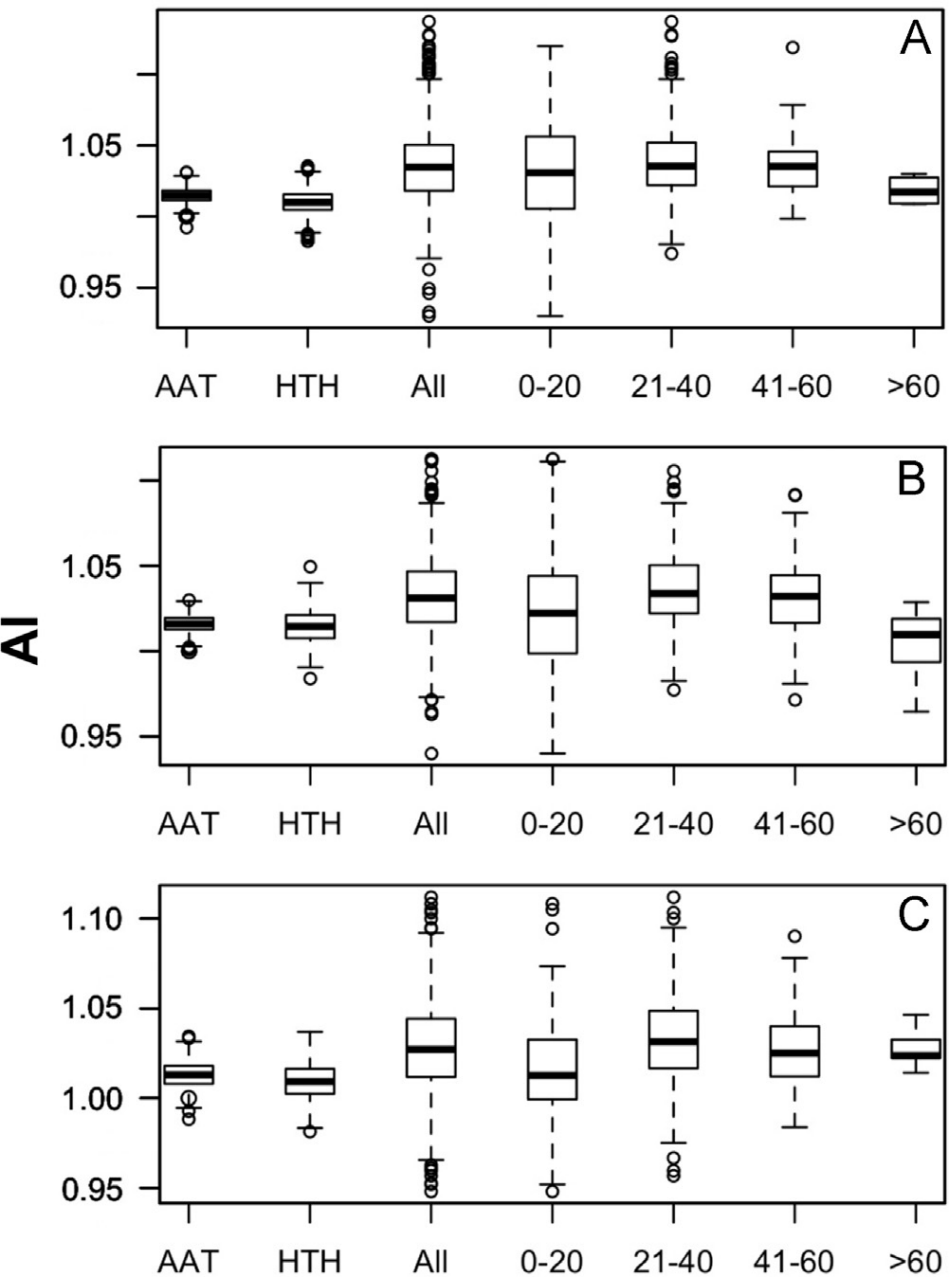
Box plots of the distribution of average Linker propensity index (#491 of Table 2 in [1] and code GEOR03010 in AAindex [10]). For interpretation of plots, refer to Fig. 6 caption.

**Fig. 9:**
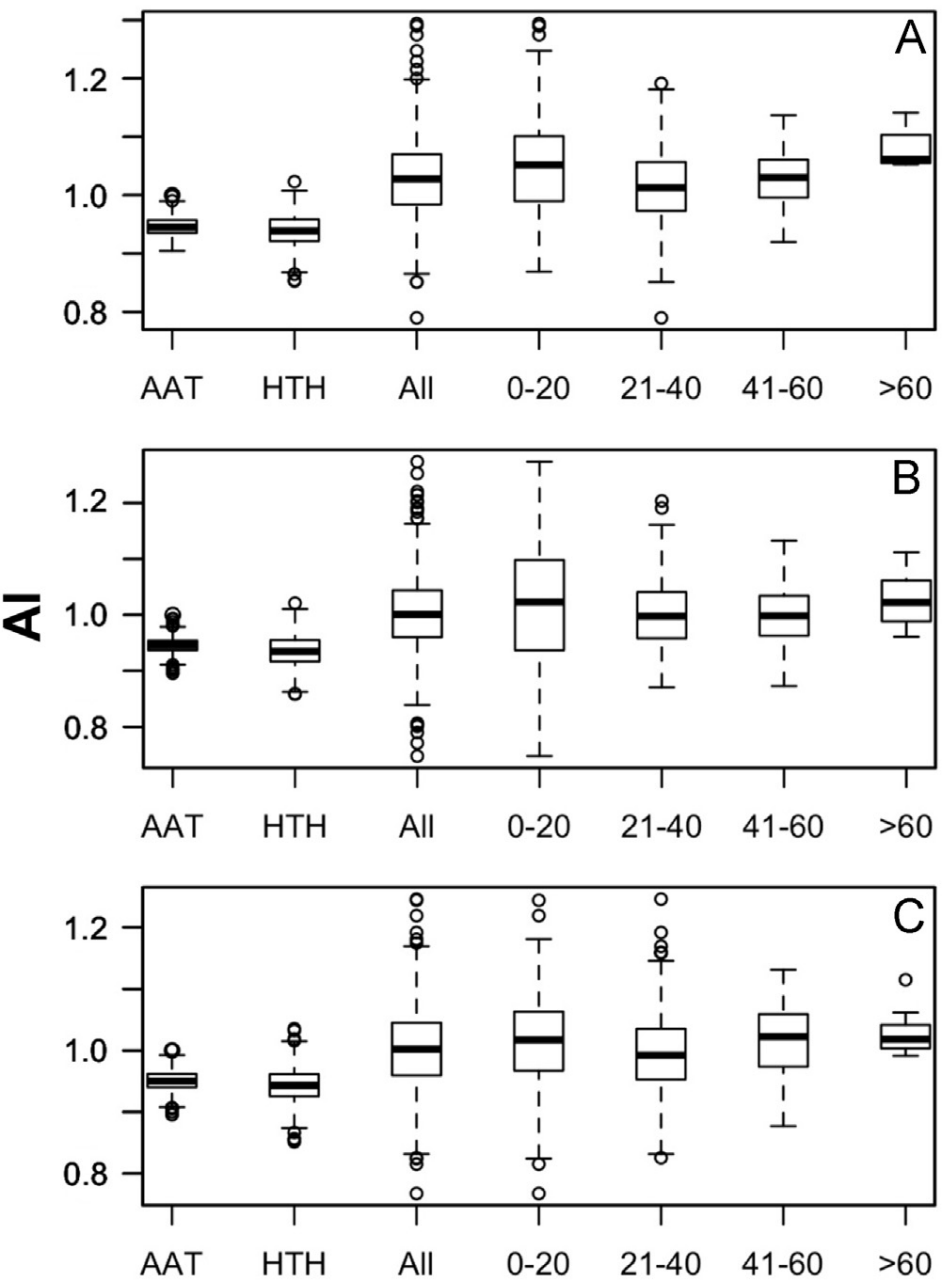
Box plots of the distribution of the average normalized β-turn propensity (index #37 Table 2 in [1] and code CHOP780101 in AAindex [10]). For interpretation of plots, refer to Fig. 6 caption.

**Fig. 10:**
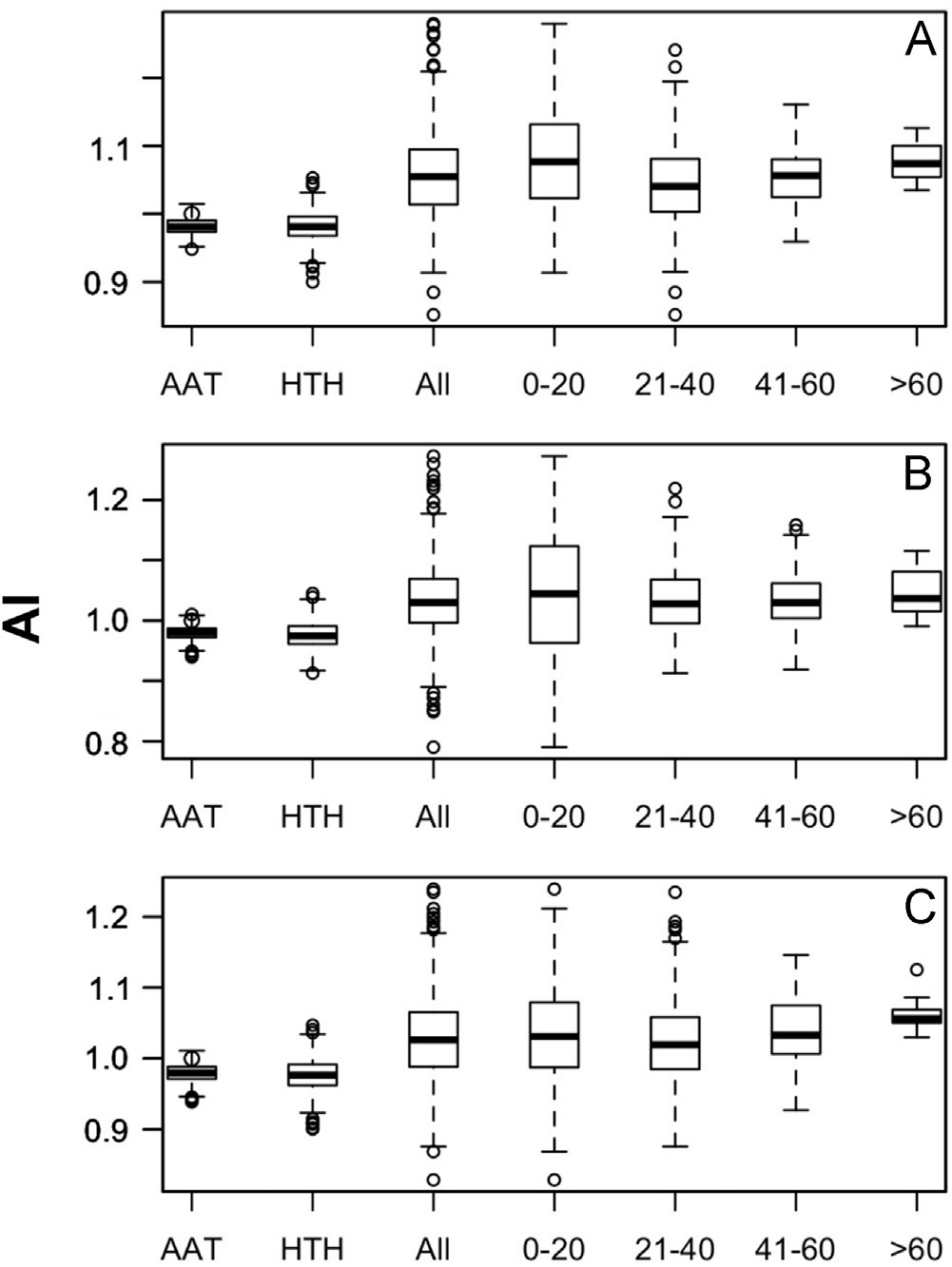
Box plots of the distribution of the average Chou–Fasman coil propensity (#24 of Table 2 in [1] and code CHAM830101 in AAindex [10]). For interpretation of plots, refer to Fig. 6 caption.

**Fig. 11:**
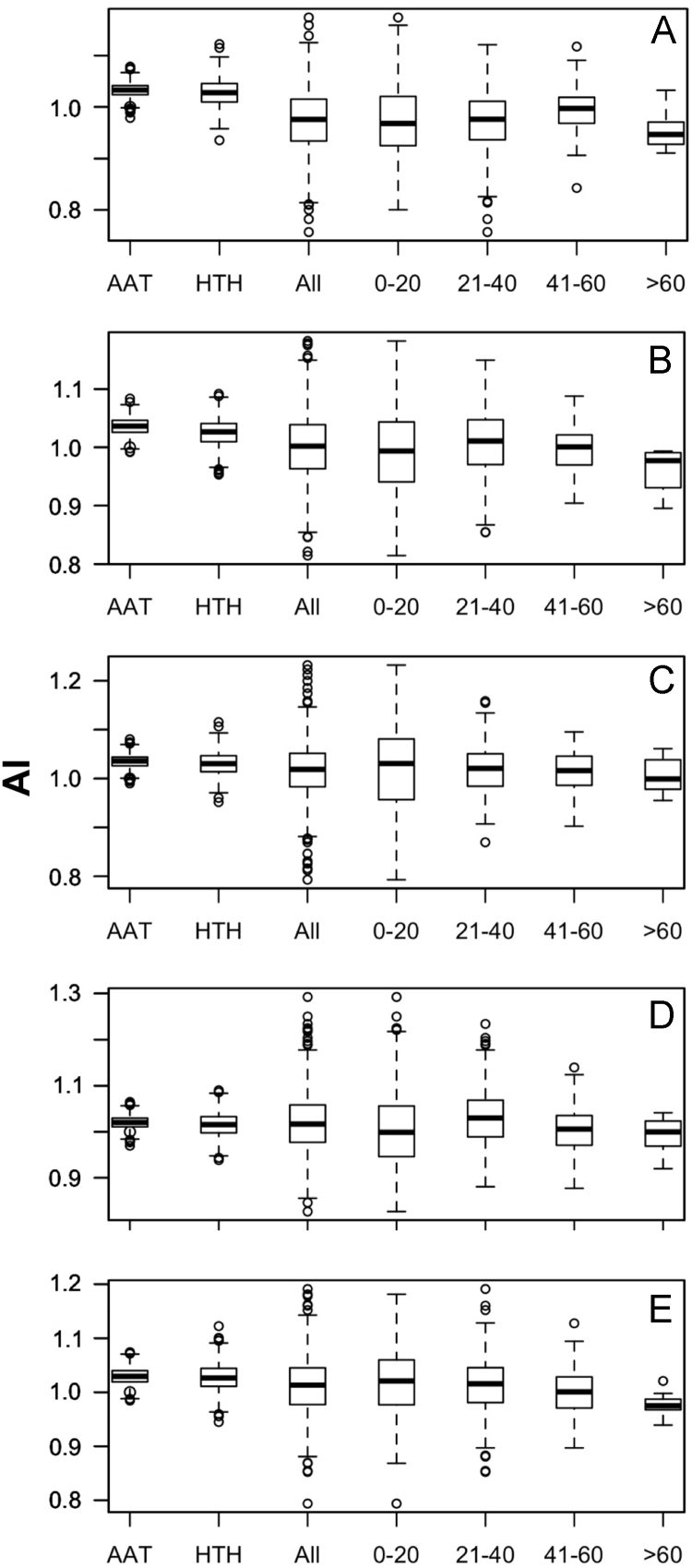
Box plots of the distribution of average normalized α-helix propensity (index #38 of Table 2 in [1] and code CHOP780102 in AAindex [10]). A, B, C, D and E denote Actinobacteria, Alphaproteobacteria, Betaproteobacteria, Firmicutes and Gammaproteobacteria, respectively.

**Fig. 12:**
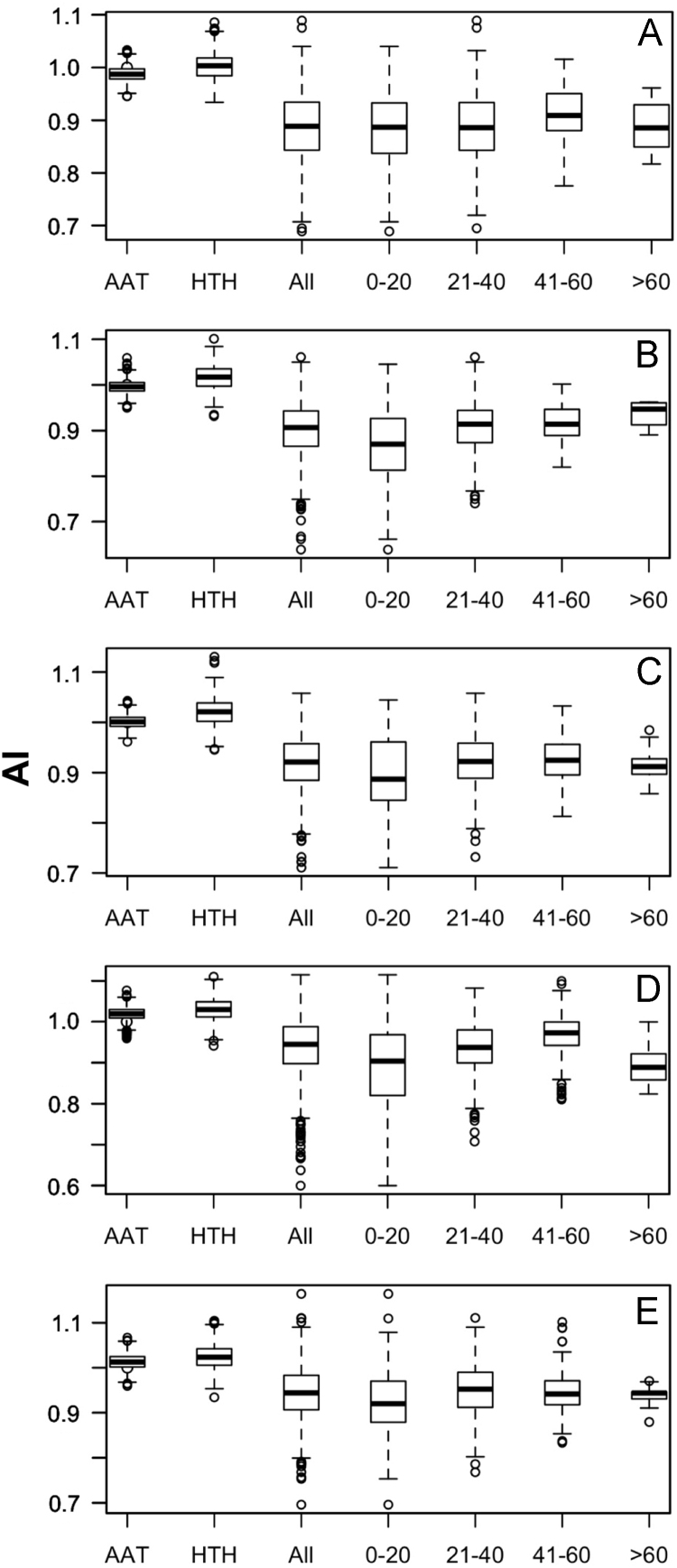
Box plots of the distribution of average normalized β-sheet propensity (index #39 of Table 2 in [1] and code CHOP780103 in AAindex [10]). Letter interpretation is as in Fig. 11 caption.

Data was created from the analysis of MocR sequences taken from the most populated phyla Actinobacteria, Firmicutes, Alpha-, Beta- and Gammaproteobacteria. Sequences of the MocR regulators in each phylum were retrieved from the UniProt data bank [2] accessed on October, 2015 with the application of RPSBLAST of the BLAST suite [3] and the CDD data bank [4]. The protein sequences containing both the wHTH and AAT domains identified by RPSBLAST were considered genuine MocR regulators. Before further processing, retrieved sequences were filtered at 75% sequence identity with the program CD-HIT [5]. Multiple sequence alignments were calculated with the programs ClustalO [6] and processed with the software Jalview [7]. Linker sequences were manually extracted from the multiple sequence alignments according with the wHTH and AAT domain boundaries assigned by RPSBLAST. List of the MocR regulators possessing linkers longer than 60 residues is reported in Table 1. Residue frequency and propensities were calculated as described in [1] and are displayed in Table 2, Table 3, Table 4, Table 5 organized according to linker length and phylum class. Propensities for the entire linker set are reported in [1]. Dipeptide frequency and propensity calculations relied on the software ‘compseq’ of the EMBOSS suite [8]. Table 6 reports the average number of residue dyads in each group. The highest the number, the highest the reliability of the dyad propensities reported in Fig. 1, Fig. 2, Fig. 3, Fig. 4, Fig. 5. Average content of predicted secondary structures (obtained with the program PREDATOR [9]) are displayed in Table 7. Physicochemical properties were assigned to the amino acid residues according to the indices provided by the AAindex data bank [10] incorporated in the Interpol package [11] of the R-project library [12]. Distribution of the properties are reported as box-plots in Fig. 6, Fig. 7, Fig. 8, Fig. 9, Fig. 10 limited to the phyla Alphaproteobacteria, Betaproteobacteria and Gammaproteobacteria and in Fig. 11, Fig. 12 for all the phyla considered. Box-plots for Actinobacteria and Firmicutes missing in Fig. 6, Fig. 7, Fig. 8, Fig. 9, Fig. 10 are to be found in [1].

**Fig. 13:**
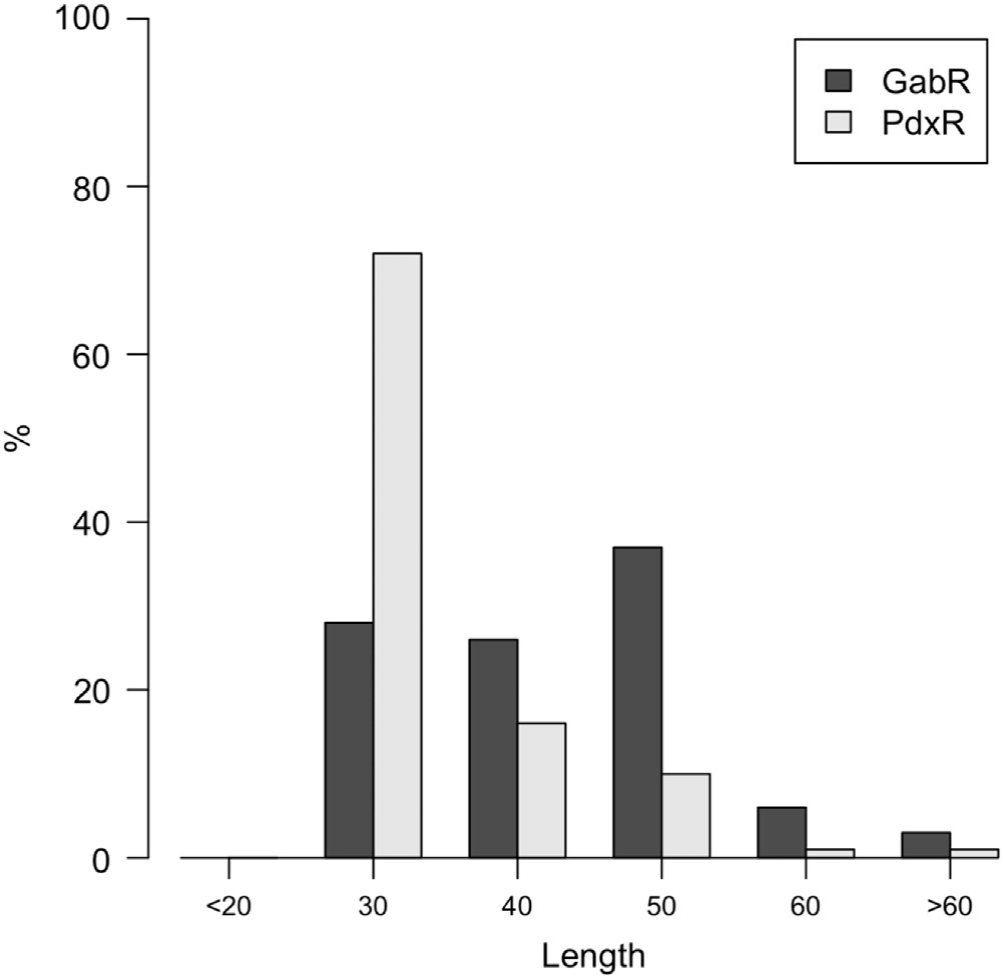
Histogram of the linker length distribution in the MocR subgroups GabR and PdxR. Horizontal axis labels indicate length intervals: 20 corresponds to 0–20, 30 (21–30), 40 (31–40), 50 (41–50), 60 (51–60) and >60 (longer than 60 residues). Percentage (%) on the vertical axis indicates the fraction of linkers in the length interval. Sequences were retrieved from the reference proteomes data bank available at the Hmmer web server [17] using a significance E-value thresholds equal to 10^−120^. With this threshold, 885 and 334 sequences were retrieved for GabR and PdxR, respectively.

The linker length distribution were analyzed within two specific MocR subfamilies: GabR [13] and PdxR [14] involved in the regulation of the synthesis of acid γ-amino butyric and pyridoxal 5′-phosphate, respectively. Sequences assigned to each of the two subgroups were retrieved from the RegPrecise data bank [15] and aligned separately (Table 8); a HMM profile [16] was calculated for each one of the multiple alignment. The profile was utilized to search for other putative GabR or PdxR sequences in the reference proteomes data bank available at the Hmmer web server [17]. Sequences showing an E-value smaller than 10^−120^, were retrieved and multiply aligned. Linker sequences were extracted as described above. Length distribution were plotted and compared for the GabR and PdxR sets (Fig. 13).

Perl and R-scripts were written for data analysis, processing and display.

## Figures and Tables

**Table 1:** List of MocR regulators predicted to have linkers of length equal or greater to 60 residues.

**UniProt code**	**Start**^a^	**End**^b^	**Length**
A0A023C4T7_9PSED	88	148	60
A0A0B2AVS1_9ACTN	85	145	60
A0NP21_LABAI	80	140	60
I9W6R0_9RALS	87	147	60
W4CMK3_9BACL	121	181	60
A0A074LC92_PAEPO	82	143	61
I4N7I5_9PSED	87	148	61
A0A0D5NE20_9BACL	85	147	62
F8FPR4_PAEMK	106	168	62
G8QJ34_DECSP	85	147	62
V7DIJ8_9PSED	88	150	62
W4P2V0_9BURK	87	149	62
B9QZW6_LABAD	80	143	63
F3KUT6_9BURK	118	181	63
F7T5G0_9BURK	85	148	63
M2X958_9MICC	80	143	63
R9LS02_9BACL	83	146	63
S2WJB8_DELAC	89	152	63
A0A098SWK7_9PSED	88	152	64
A0A0J6J2M6_9PSED	88	152	64
A0A0F4KHT0_9ACTN	101	166	65
D5BN74_PUNMI	82	147	65
D7DQ74_METV0	90	156	66
K0YXF4_9ACTN	79	145	66
A0A077LFC1_9PSED	87	154	67
A0A095YU49_9FIRM	78	145	67
H0BWG7_9BURK	75	142	67
A0A087DUC1_9BIFI	78	146	68
A0A090ZGE9_PAEMA	83	152	69
A0A0A6Q9N6_9BURK	74	143	69
F3JEN8_PSESX	88	157	69
W0HH53_PSECI	88	157	69
A0A0A6QBJ9_9BURK	89	159	70
A0A0B4DLS5_9MICC	89	159	70
A0A088Y9M0_BURPE	88	159	71
A0A0F4JB47_9ACTN	62	135	73
A0A069DE36_9BACL	85	159	74
A0A087EGV8_9BIFI	105	181	76
A0A089I7M0_9BACL	82	158	76
A8SVX0_9FIRM	79	155	76
A0A089N895_9BACL	78	155	77
A0A0F5JX35_9BURK	84	161	77
A0A0E4CZM5_9BACL	90	168	78
A0A061LXN0_9MICO	84	163	79
A0A0A8BLT7_9BURK	89	168	79
R6HHE8_9ACTN	79	159	80
X4ZGS7_9BACL	84	164	80
A0A089HPN9_PAEDU	78	162	84
D2PX75_KRIFD	93	178	85
D3F8U9_CONWI	80	166	86
A0A0A4HID4_9PSED	88	179	91
F2RK57_STRVP	86	180	94
C7MPD0_CRYCD	79	174	95
F4QXL0_BREDI	83	179	96
A0A087AB73_9BIFI	78	175	97
A0A087E7D4_9BIFI	78	175	97
A0A0B4DPH0_KOCRH	85	183	98
F2RA50_STRVP	88	186	98
V6KRX5_STRRC	93	191	98
M8D4I1_9BACL	79	179	100
A0A0A3JRX6_BURPE	88	189	101
M8DED6_9BACL	80	183	103
A0A087BLK1_BIFLN	78	187	109
A0A087CXD8_9BIFI	78	187	109
S6CDU1_9ACTN	130	244	114
A0A0A6SYE7_9BURK	87	209	122
F5LR05_9BACL	82	209	127
A0A089IZ38_PAEDU	84	218	134
A0A0B6S8F7_BURGL	88	231	143
A0A087A119_9BIFI	78	222	144
A0A089MC10_9BACL	82	234	152
A0A089KZI8_9BACL	82	244	162

a Linker N-terminal sequence position.

b Linker C-terminal sequence position.

**Table 2:** Residue propensities in the linkers of length range 0–20.

^a^Amino acid one-letter code.

^b^Residue propensity; cells containing values ≥1.01 and ≤1.19 and values ≥1.20 are shaded with light and dark grey respectively. In the latter case, numbers are boldfaces.

^c^Number of residues in the sample.

**Table 3:** Residue propensities in the linkers of length range 21–40.

^a^Amino acid one-letter code.

^b^Residue propensity; cells containing values ≥1.01 and ≤1.19 and values ≥1.20 are shaded with light and dark grey respectively. In the latter case, numbers are boldfaces.

^c^Number of residues in the sample.

**Table 4:** Residue propensities in the linkers of length range 41–60.

^a^Amino acid one-letter code.

^b^Residue propensity; cells containing values ≥1.01 and ≤1.19 and values ≥1.20 are shaded with light and dark grey respectively. In the latter case, numbers are boldfaces.

^c^Number of residues in the sample.

**Table 5:** Residue propensities in the linkers of length range 61–200.

^a^Amino acid one-letter code.

^b^Residue propensity; cells containing values ≥1.01 and ≤1.19 and values ≥1.20 are shaded with light and dark grey respectively. In the latter case, numbers are boldfaces.

^c^Number of residues in the sample.

**Table 6:** Average number of residue pairs in each data set.

	**Length intervals**				
	**All**	**0–20**	**21–40**	**41–60**	**61–200**
**Actinobacteria**	53.5±93.1	9.2±17.6	29.2±53.8	10.0±16.4	5.0±8.5
**Alphaproteobacteria**	45.7±56.7	6.0±9.0	20.3±28.5	18.9±22.4	0.5±0.8
**Betaproteobacteria**	57.1±78.2	3.2±5.1	25.5±35.1	25.1±34.8	3.0±5.8
**Firmicutes**	83.0±63.5	6.4±6.8	39.9±34.8	32.4±25.0	4.4±6.4
**Gammaproteobacteria**	82.0±81.9	8.7±9.4	50.8±54.1	20.9±20.6	1.5±3.5

**Table 7:** Fraction of predicted secondary structure in linker regions.

	**Secondary structure**		
	**α-helix**	**β-strand**	**coil**
**Actinobacteria**	0.14	0.02	0.86
**Alphaproteobacteria**	0.19	0.03	0.78
**Betaproteobacteria**	0.30	0.01	0.69
**Firmicutes**	0.02	0.06	0.92
**Gammaproteobacteria**	0.26	0.02	0.72

**Table 8:** GabR and PdxR sequences retrieved from RegPrecise data bank.

**GabR**		
**UniProt code**	**Specie**	**Phylum**
A0A098SFD5	*Acinetobacter baumannii* AB0057	*Gammaproteobacteria*
Q6F766	*Acinetobacter sp*. AD	*Gammaproteobacteria*
A7Z1D7	*Bacillus amyloliquefaciens* FZB42	*Firmicutes*
A8F9Y9	*Bacillus pumilus* SAFR 032	*Firmicutes*
P94426	*Bacillus subtilis subsp. subtilis str*. 168	*Firmicutes*
Q2KX56	*Bordetella avium* 197N	*Betaproteobacteria*
A0A0H3LKN1	*Bordetella bronchiseptica* RB50	*Betaproteobacteria*
Q0B6G3	*Burkholderia cepacia* AMMD	*Betaproteobacteria*
C5ALU9	*Burkholderia glumae* BGR1	*Betaproteobacteria*
A0A0H2XDM4	*Burkholderia mallei* ATCC 23344	*Betaproteobacteria*
B2JSD8	*Burkholderia phymatum* STM815	*Betaproteobacteria*
B2JR38	*Burkholderia phymatum* STM815	*Betaproteobacteria*
Q63NL7	*Burkholderia pseudomallei* K96243	*Betaproteobacteria*
A4JJX2	*Burkholderia vietnamiensis* G4	*Betaproteobacteria*
Q13LC0	*Burkholderia xenovorans* LB400	*Betaproteobacteria*
A9BMY2	*Delfia acidovorans* SPH-1	*Betaproteobacteria*
D4HXE9	*Erwinia amylovora* ATCC 49946	*Gammaproteobacteria*
Q6D5I8	*Erwinia carotovora subsp.atroseptica* SCRI1043	*Gammaproteobacteria*
A6TF79	*Klebsiella pneumonia subsp. pneumoniae* MGH 78578	*Gammaproteobacteria*
B2U7Y5	*Ralstonia pickettii* 12J	*Betaproteobacteria*
A8GJW1	*Serratia proteamaculans* 568	*Gammaproteobacteria*
Q4A0R1	*Staphylococcus saprophyticus subsp. saprophyticus* ATCC 15305	*Firmicutes*
C4ZIR5	*Thauera sp.*MZ1T	*Betaproteobacteria*
Q7CJK7	*Yersinia pestis* KIM	*Gammaproteobacteria*
A1VQK3	*Polaromonas naphthalenivorans* CJ2	*Betaproteobacteria*
Q129G7	*Polaromonas sp.* JS666	*Betaproteobacteria*
Q221G1	*Rhodoferax ferrireducens* DSM 15236	*Betaproteobacteria*
C5CM40	*Variovorax paradoxus* S110	*Betaproteobacteria*

**PdxR**		
B9MKZ0	*Anaerocellum thermophilum* DSM6725	*Firmicutes*
A4XIB4	*Caldicellulosiruptor saccharolyticus* DSM 8903	*Firmicutes*
Q929S0	*Listeria innocua* Clip11262	*Firmicutes*
Q8Y5G3	*Listeria monocytogenes* EGD e	*Firmicutes*
A0AKK7	*Listeria welshimeri serovar 6b str.* SLCC5334	*Firmicutes*
C7MF20	*Brachybacterium faecium* DSM 4810	*Actinobacteria*
Q6AFC0	*Leifsonia xyli subsp.xyli str*. CTCB07	*Actinobacteria*
B3GXB5	*Actinobacillus pleuropneumoniae servar 7 str.* AP76	*Gammaproteobacteria*
Q5WKW3	*Bacillus clausii* KSM K16	*Firmicutes*
C3PLB2	*Corynebacterium aurimucosum* ATCC 700975	*Actinobacteria*
Q6NK11	*Corynebacterium diphtheriae* NCTC 13129	*Actinobacteria*
Q8NS92	*Corynebacterium glutamicum* ATCC 13032	*Actinobacteria*
B2GK63	*Kocuria rhizophila* DC2201	*Actinobacteria*
B9E8T3	*Macrococcus caseolyticus* JCSC5402	*Firmicutes*
W8TRW2	*Staphylococcus aureus subsp. aureus* N325	*Firmicutes*
B9DKX6	*Staphylococcus aureus subsp.carnosus* TM300	*Firmicutes*
A0A0H2VKR4	*Staphylococcus epidermidis* ATCC 12228	*Firmicutes*
A0A0Q1AKJ7	*Staphylococcus haemolyticus* JCSC1435	*Firmicutes*
Q49V27	*Staphylococcus saprophyticus subsp.saprophyticus* ATCC15035	*Firmicutes*
