# Supplementary material for: Data from computational analysis of the peptide linkers in the MocR bacterial transcriptional regulators
Source: Data Brief. 2016 Sep 5;9:292–313. doi: 10.1016/j.dib.2016.08.064 (PMC5026710; doi:10.1016/j.dib.2016.08.064)
Supplement: Supplementary file 1 — Supplementary material [file mmc1.doc]

Rome, August 16 th, 2016

Data in Brief

Editorial Office

We wish to confirm that there are no known conflicts of interest associated with this publication and there has been no significant financial support for this work that could have influenced its outcome

On behalf of all the authors,

Stefano Pascarella
